# Investigating Avian Neglect and Welfare Violations: Case Studies in South Korea

**DOI:** 10.1155/crve/6512939

**Published:** 2025-03-05

**Authors:** Goun Park, JaeKyeom Kim, Chunghyun Kim

**Affiliations:** ^1^Department of Animal Health and Welfare, College of Life and Health Sciences, University of Hoseo, Asan-si, Republic of Korea; ^2^Avian Disease Division, Animal and Plant Quarantine Agency, Gimcheon-si, Republic of Korea

**Keywords:** animal welfare, botulism, methomyl poisoning, ostrich, quail

## Abstract

This paper investigates three significant cases of avian welfare violations in South Korea, emphasizing the detrimental effects of neglect and inadequate management practices. The first case concerns the mass mortality of birds in a Seoul park due to methomyl poisoning. The second case focuses on a botulism outbreak occurring on a quail farm. The third case examines the sustained mortality resulting from nutritional deficiencies and environmental stress on an ostrich farm. These cases highlight the urgent need for improved animal welfare practices and stricter regulatory measures to ensure the protection and well-being of avian species.

## 1. Introduction

Animal welfare has emerged as a critical global issue, increasingly influencing societal attitudes and policies in many regions [1]. In South Korea, rapid urbanization and the expansion of the pet market have heightened awareness of animal welfare concerns [2]. This cultural evolution is typified by the passage of the dog meat consumption ban law in 2020, which faced minimal public opposition and marks a significant shift in societal perceptions of animal rights and welfare [3].

Despite positive developments surrounding companion animals, considerable disparities persist in public understanding of welfare issues concerning farm animals [4]. Many citizens remain largely unaware of the harsh conditions livestock frequently endure, including pervasive mistreatment within agricultural practices [4]. The 2017 pesticide egg scandal served as a notable wake-up call, exposing critical deficiencies in animal welfare standards within the poultry industry and igniting national discussions on the ethical treatment of farm animals [5]. In response to these revelations, the South Korean government has begun implementing various animal welfare certifications for major livestock species, including cattle, pigs, chickens, and ducks [5]. Nevertheless, avian welfare, particularly for farmed birds, has yet to be thoroughly addressed, revealing a significant gap in regulatory measures and public awareness.

This paper is aimed at exploring the three notable cases of animal welfare violations involving birds, analyzing the contextual factors surrounding each incident and their broader implications for animal welfare policy in South Korea. By examining these instances, this study seeks to highlight the gravity of neglect experienced by affected birds and the significant role of public perception in shaping animal rights and welfare dialogues.

## 2. Case 1: Methomyl Poisoning in Wild Birds

On July 22, 2020, at approximately 01:30 AM, the Seoul Mapo Police Department received a tip of a mass mortality event involving numerous wild birds at the plaza near Exit 1 of Sogang University Station in Nogosan-dong, Mapo-gu, Seoul. Upon arrival, the responding officers confirmed the death of approximately 100 wild birds at the scene. Among the deceased birds, 11 specimens were selected for further analysis, consisting of nine pigeon carcasses and two sparrow carcasses. Additionally, rice grains discovered in close proximity to the carcasses were gathered and submitted for analysis to investigate any potential link to the birds' deaths. These samples were submitted to the Animal and Plant Quarantine Agency for disease investigation to determine the potential causes of this mortality event. It was noted that Sogang University Station has a daily ridership of approximately 20,000 passengers. As a result, this incident has created a concerning situation for local residents, and regional media are also closely monitoring the developments surrounding this case.

We initiated a necropsy process, considering the simultaneous deaths of over 100 birds in a park with significant human contact. This raised concerns about potential exposure to acute zoonotic diseases such as avian influenza or botulism and other infectious diseases or toxicological poisoning among avian populations.

**Figure 1:**
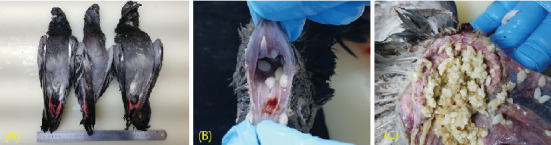
(A) Mass mortality due to methomyl poisoning affected pigeons in a park. (B) Petechial hemorrhage in the oral mucosa. (C) Proventriculus contained a significant number of fresh rice grains.

During the external examination of all 11 birds, the observed weights approached values typical of normal individuals. Upon conducting the necropsy, the birds exhibited minimal resistance during evisceration, suggesting an absence of dehydration. Notably, a substantial quantity of fresh rice grains was identified within the proventriculus and ventriculus. These observations collectively indicate that the birds were in a relatively healthy state with a good appetite prior to their sudden mortality. Furthermore, the exceptional freshness of the rice grains implies a significant correlation between the presence of these grains and the abrupt deaths, warranting further investigation into potential toxicological or environmental factors contributing to this event (Figure 1).

Among the 11 birds, one showed pathological lesions of petechial hemorrhage in the oral mucosa; however, no signs of necrosis or hemorrhage were detected in the internal organs of the remaining birds. The samples from the dead birds were used for testing avian influenza, Newcastle disease, and bacterial diseases according to the manual of the Animal and Plant Quarantine Agency's Avian Disease Division. For toxicological analysis, the stomach contents of the necropsied animals and the suspected rice grains as feed were sent to the National Forensic Service.

Testing for acute infectious disease, avian influenza, Newcastle disease, and bacterial infections was conducted, but all results were confirmed to be negative. The National Forensic Service examined the submitted samples, focusing on seven specific substances: cyanide, organophosphates, organochlorines, carbamates, propylene glycol, eugenol, and methomyl. The findings indicated negative results for cyanide, organophosphates, organochlorines, carbamates, propylene glycol, and eugenol. However, the presence of methomyl in the stomach contents of the necropsied animals and the suspected rice grains used as feed was definitively confirmed through physicochemical testing and gas chromatography–mass spectrometry (GC-MS) techniques.

Based on the detection of methomyl in both the feed and the stomach contents, it is hypothesized that the mass mortality of wild birds in the park may be attributed to an individual who intentionally mixed methomyl with rice grains to poison the birds. In response to this finding, the Mapo Police Department collected CCTV footage from the park on the day of the incident, which revealed video evidence of a man in his 70s feeding the birds. After questioning, the suspect confessed to intentionally spreading rice grains laced with methomyl throughout the park due to his frustrations over bird droppings during his walks. This incident illustrates animal abuse resulting from individuals' emotional distress.

Methomyl is an extremely toxic organophosphate pesticide, and even minimal exposure can lead to severe intoxication and fatal outcomes due to its potent neurotoxic properties. It is rapidly absorbed through the skin and respiratory system, affecting the nervous system and complicating treatment. Additionally, the odorless and colorless nature of methomyl makes detection challenging, increasing the risk of accidental exposure.

## 3. Case 2: Outbreak of Botulism in Quail

In September 2004, a random act of crime occurred at Dalseong Park in Daegu, South Korea, where individuals were poisoned by yogurt contaminated with methomyl. The first victim was a 63-year-old homeless man who consumed yogurt left on a bench, dying within 2 h from acute poisoning. The victims included a diverse group of individuals such as the elderly, children, and homeless people, with a total of 14 people affected in this incident. Due to the lack of substantial leads to apprehend the perpetrator, the case remains unsolved to this day. In a related context, on February 20, 2013, six elderly individuals died after consuming bean sprout rice at a restaurant in Cheongbuk Boeun; both incidents involved yogurt and soy sauce mixed with methomyl.

The South Korean government responded to these concerns by revoking methomyl's registration in 2011 and banning its use nationwide starting in 2015. The suspect confessed to secretly purchasing it through illicit “under-the-table” transactions, as it was sold at a premium due to its effective pest control properties [6].

Comparative analysis reveals that the recent methomyl poisoning incident in Seoul is similar to other incidents occurring in different countries, including the poisoning of peregrine falcons in Zurich, Switzerland, and significant mortality events in Medellin, Colombia [7, 8]. In Zurich, surveillance footage documented the deaths of peregrine falcons after consuming methomyl-laced pigeons, further raising concerns about the illegal killing of wildlife [8]. Similarly, in Medellin, hundreds of pigeons died due to methomyl exposure, with necropsies revealing that their crops contained tainted corn kernels [7].

In summary, this incident constitutes a violation of South Korea's Animal Protection Act, which unequivocally forbids the infliction of harm on animals through the application of toxic substances [9]. The intentional administration of methomyl, a pesticide that has been officially banned in South Korea since 2015 due to its pronounced toxicity and potential for harmful misuse, represents a blatant infringement of the legal safeguards established to protect animal welfare. Such actions not only contravene the legislative framework designed to ensure the ethical treatment of animals but also underscore the urgent need for enhanced enforcement mechanisms to prevent similar incidents in the future.

On August 9, 2022, a call was received at the Animal and Plant Quarantine Agency from a woman in her 60s who had been operating a quail farm for over 10 years. She indicated that since the conclusion of the monsoon season in July, the farm had experienced a mortality rate of 200 to 300 quails per day over the course of a month. She noted that during the monsoon season, the mortality rate increases, and there is a tendency for it to decrease during the autumn and winter months. This phenomenon has persisted continuously over the past 5 years, causing significant challenges in livestock management. These ongoing issues prompted her to consult the Gyeongbuk Research Institute; however, no definitive cause has been identified.

In response to the ongoing situation, an investigation team visited the quail farm on August 10, 2022, for further evaluation. The quail farm had three houses, with 100,000 birds in House 1, 100,000 in House 2, and 50,000 in House 3. The houses had separate entrances but no partitions between them. The quails were over 30 weeks old. Each cage measured 50 × 40 cm and housed 30 birds in a 7-tier arrangement. The appropriate stocking density is 75 cm^2^ per bird, but the farm in question was raising five more birds per cage than the recommended amount.

The feed supplier visited approximately four times a week, and vaccinations against diseases such as Newcastle disease were not administered. Within 300 m, there was a cattle farm with about 50 heads of cattle, and both farms used the same road to access their properties. The biosecurity facilities included an external parking lot, fencing, entrance blocking facilities, vehicle disinfection equipment, and wild bird barriers; however, it did not have a goods intake warehouse or a disinfection room.

**Figure 2:**
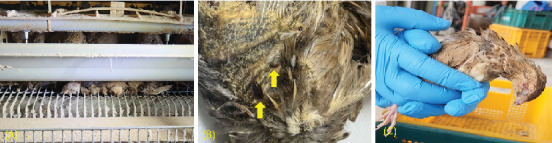
(A) Dead carcasses infected with botulism are raised alongside live quails in cages. (B) Maggot and insect on the dead quail surface (arrow). (C) Quail with clinical signs of neck paralysis.

During the on-site inspection, mist spraying was utilized to mitigate elevated temperatures; however, the ventilation within the facility was observed to be insufficient. In the severely affected cages, only 15 out of the original 30 birds were present, either alive or deceased. Among the 10 birds that remained alive, two were found lying on the floor in a state of near death. Additionally, five carcasses had been left in the cage without removal. Some of these deceased birds were noted to have two to three maggots and insects present, and several carcasses exhibited external damage (Figure 2). Severely affected birds were either deceased or displayed significant lethargy and weakness, lying prostrate on the floor, while healthy birds in the same cage were observed pecking at the lethargic or deceased individuals. The highest mortality rates were recorded in the cages located on the third to fifth tiers, which are easily accessible to workers. From the group of severely affected individuals, some were extracted from the barn for clinical observation. The clinical signs recorded included paralysis of the legs, wings, and neck, along with flaccid paralysis and diarrhea. For diagnostic purposes, samples were meticulously collected, including carcasses (20 birds), larvae (6), gloves (3), feed (3), groundwater (2), cages (6), and shoes (6).

Upon returning to the laboratory, we performed necropsies on the 20 birds that exhibited clinical signs or were found deceased. Our examination revealed no evidence of hemorrhagic lesions in internal organs such as the respiratory, reproductive, muscular, or skeletal systems attributable to viral or bacterial infections. Notably, loose feces were observed in the intestinal tract. The samples from the dead birds were used for testing avian influenza, Newcastle disease, and bacterial diseases according to the manual of the Animal and Plant Quarantine Agency's Avian Disease Division.

Additionally, due to suspected botulism, we conducted specific assays to detect the presence of botulinum toxins. The tests for botulism were conducted according to the standard diagnostic procedures for animal diseases at the Animal and Plant Quarantine Agency, including polymerase chain reaction (PCR), bacterial isolation, and mouse inoculation. The diagnostic method involved mouse bioassays using serum samples from botulism-affected birds and tryptone-peptone-glucose-yeast extract (TPGY) inoculums. Serum or supernatants were injected into ICR mice to observe symptoms of botulism. Surviving mice were euthanized for further testing with *Clostridium botulinum* antitoxins. PCR was performed on DNA extracted from cecal contents and livers to detect botulinum toxin genes using specific primer pairs. *C. botulinum* was isolated from organ material inoculated into the TPGY broth and cultured anaerobically [10].

All diagnostic assays, with the exception of botulism testing, yielded negative results. The results, including PCR, bacterial isolation, and mouse inoculation, indicated positive findings for botulism in the livers and diarrheal contents of the carcasses (13 out of 20), as well as in the workers' gloves (6 out of 6), cages (4 out of 6), and maggot samples (4 out of 6). However, the feed, shoes, and groundwater samples tested negative. We concluded that the cause of the mass mortality in the quail population was botulism poisoning.

Insufficient workforce numbers delayed the removal of dead birds, contributing to contamination. The failure to promptly separate the deceased quails from the cage during the initial outbreak was crucial to the outbreak's severity, allowing fly larvae to proliferate and act as vectors for the toxin. Fly infestations and cannibalism among the quails exacerbated the transmission of botulinum toxin. An infection case caused by fly larvae also occurred in late summer 2010 among birds kept in a dam at a southern Brazilian zoo [11].

Birds can acquire botulism through direct ingestion of the toxin or by consuming contaminated food. They often pick up the bacteria by feeding on infected invertebrates. This creates a cycle where dead animals and warm temperatures attract flies, which lay eggs that hatch into maggots. These maggots feed on birds that have succumbed to botulism, accumulating the toxin in their bodies. When other birds consume these maggots, they may also fall ill or die, thereby amplifying the spread of the disease. It is noteworthy that birds can develop botulism after consuming just a few larvae [12].

The high mortality rates observed between the third and fifth tiers are believed to have been exacerbated by the spread of the disease through the workers' gloves. During the process of removing deceased birds from the cages, the gloves likely became contaminated, which could have led to the continuous contamination of adjacent cages during feed provision and cleaning activities with the contaminated gloves.

The outbreak of botulism at the quail farm highlights significant concerns regarding animal welfare and farm management. The primary contributing factors included inadequate carcass removal due to labor shortages, bacterial proliferation and larval infestations resulting from insufficient ventilation, and stress associated with overcrowding, which led to cannibalism. This overcrowding not only compromised the welfare and health of the animals but also spurred aggressive behaviors such as cannibalism, which likely exacerbated the spread of the botulinum toxin. The overwhelming mortality rate was largely attributed to management negligence and inadequate environmental conditions.

Instructions were given to promptly remove deceased carcasses from the cages, avoid mist spraying to increase humidity, refrain from overcrowding, and implement measures to control flies and larvae. Post intervention, the mortality rate diminished, reinforcing the importance of maintaining stringent animal welfare standards. This case illustrates the critical need for farms to adopt comprehensive protocols that ensure the health and welfare of farmed animals while emphasizing the consequences of neglect.

In summary, this case exemplifies critical breaches of animal welfare standards arising from inadequacies in the implementation of appropriate husbandry practices [9]. The failure to uphold humane stocking densities and ensure adequate hygienic conditions resulted in profound adverse effects on the health and welfare of the livestock, culminating in elevated mortality rates. This situation underscores the pressing necessity for enhanced regulatory oversight and the establishment of robust management protocols within the agricultural industry.

## 4. Case 3: Gastrointestinal Impaction in Ostrich

On October 19, 2022, continuous mortality among the ostrich population at a farm in Andong was reported, where three out of a total of 31 ostriches had died. In one of the rearing sheds, a 1.5-year-old ostrich died on October 13, followed by another on October 17, while a 10-year-old ostrich passed away in a separate shed. In response, the farm owner solicited a disease investigation from the Animal and Plant Quarantine Agency, which prepared the necessary necropsy tools and conducted an on-site evaluation.

**Figure 3:**
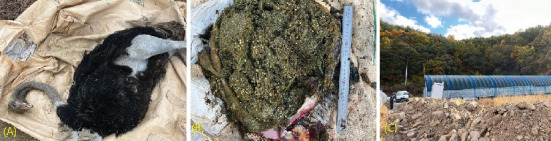
(A) Death of an ostrich was caused by gastrointestinal impaction. (B) Significant amounts of sand and rice husks were found in the ostrich's stomach. (C) Construction site was suspected of causing noise-induced stress around the farm.

The farm owner reported that the ostriches exhibited signs of reduced appetite and vitality about 1 week prior to the deaths. Speculation arose that recent cold weather may have contributed to the mortality. There were no other livestock farms within a 1-km radius of the animal farm, and the birds were raised both indoors and outdoors. An ongoing road construction project, approximately 300 m away, involved an average of 20 dump trucks entering the premises daily to deposit soil around the farm's perimeter (Figure 3). Furthermore, the COVID-19 pandemic disrupted regular supply chains, causing the farm to rely on discarded vegetable waste from local farmers' markets instead of higher cost commercial feed.

Necropsies revealed that the ostriches' stomachs were filled with significant amounts of sand and rice husk, indicating death due to gastrointestinal impaction. No signs of infectious diseases, including avian influenza, were identified during the examination. The pathologist concluded that the primary cause of mortality was gastrointestinal impaction resulting from sand and rice husk ingestion.

The ongoing mortality rates at the ostrich farm highlight the critical importance of adhering to animal welfare standards, particularly regarding proper nutrition and the management of environmental stressors. Inadequate welfare conditions, especially the failure to prevent hunger, have led to serious health issues and deaths among the ostriches. Stress in avian species increases their vulnerability to various negative outcomes, resulting in production losses in poultry [13].

Environmental stressors include factors such as temperature, humidity, handling practices, transportation, stocking density, noise levels, limited access to feed, social hierarchies, and the presence of nearby predators [14–17]. These factors can significantly affect the feeding behavior of ostriches and may lead to conditions like pica [18]. When birds are unable to access food due to these stressors, they may begin to peck at nonfood items, which can ultimately lead to blockages in the stomach [13, 19–22]. This situation underscores the urgent requirement for rigorous management strategies and regulatory oversight to protect the welfare of exotic animals within farming environments.

In summary, this case exemplifies a failure to adhere to es`lished animal welfare standards that mandate the provision of adequate nutrition and effective environmental management [9]. Such noncompliance is critical, as it can lead to health issues induced by stress and compromise the overall well-being of the animals involved. This situation highlights the necessity for rigorous adherence to welfare protocols to mitigate adverse health outcomes and promote optimal animal care.

## 5. Discussion

Analyzing public perception, legislation, and the realities of farm animal welfare in South Korea reveals a complex landscape that has evolved in recent years. The passage of the dog meat consumption ban in 2020 has positively influenced societal attitudes toward animal welfare, highlighting the growing importance of animal rights among citizens. However, awareness regarding the welfare of farm animals remains insufficient. Many individuals do not fully grasp the suffering and challenges faced by livestock, a gap largely attributed to inadequate media coverage and limited educational outreach.

Recent incidents showcasing violations of animal welfare—characterized by insufficient living conditions, lack of proper nutrition, and inadequate veterinary care—underscore the pressing challenges faced in South Korea [23]. These cases not only reflect a failure to uphold basic welfare standards but also raise critical ethical questions about societal responsibilities toward all sentient beings, regardless of species.

Despite the existence of legal frameworks, such as the Animal Protection Act, practical enforcement and monitoring systems are lacking in South Korea. While this legislation aims to ensure the basic welfare of animals, it often falls short due to insufficient penalties and oversight for farm animals. As a result, many measures designed to uphold animal dignity are not effectively implemented.

Resolving these issues requires more than just policy changes; there is a clear need for systems that evaluate and manage realistic animal welfare standards through active enforcement and oversight. Although various animal protection organizations have raised these concerns, government responses have been slow and inefficient.

The current realities for farm animals in South Korea are stark. Many endure severe suffering due to poor living conditions and inhumane management practices. Overcrowding is a significant issue, particularly in quail farms, where it leads to insect infestations and disease transmission, directly contradicting the principles of animal welfare. Such conditions not only harm the welfare of livestock but also pose serious risks to public health.

Inadequate management and nutrition further exacerbate these problems. For instance, ostriches are not provided with appropriate nutrition or environments conducive to natural behaviors may experience stress and exhibit abnormal behaviors.

To improve the welfare of farm animals, several measures are essential. Public education and awareness initiatives must be enhanced to foster a deeper understanding of animal welfare among citizens. This can stimulate societal discussions about animal rights and welfare. Additionally, strengthening policy and legal frameworks is crucial, with an emphasis on improving monitoring and enforcement to ensure the effectiveness of existing laws.

Moreover, ongoing education and resources should be provided to farm managers to facilitate compliance with animal welfare standards, ultimately leading to better management and treatment of animals.

By adopting these systematic approaches, South Korea can more effectively address the challenges related to farm animal welfare. Recognizing animal welfare as not only a legal issue but also a matter of social ethics and responsibility will be vital in fostering a more humane treatment of all animals.
